# The Use of Tutomesh for a Tension-Free and Tridimensional Repair of Uterovaginal and Vaginal Vault Prolapse: Preliminary Report

**DOI:** 10.1155/2015/303679

**Published:** 2015-09-06

**Authors:** Danilo Dodero, Luca Bernardini

**Affiliations:** ^1^Divisione di Ostetricia e Ginecologia, ASL 4 Chiavarese, Ospedale Rivoli, 16033 Lavagna, Italy; ^2^Dipartimento Materno Infantile, Ostetricia e Ginecologia, ASL 5 Spezzino, 19100 La Spezia, Italy

## Abstract

*Objective*. To evaluate efficacy in terms of vaginal capacity, coital function, and recurrence prevention of a new biological mesh of bovine pericardium (Tutomesh) in the repair of severe POP.* Methods*. Thirty cases of patients suffering from stage III uterine or apical prolapse undergone surgical repair by means of a modified sacrospinous ligament suspension combined with mesh attachment to both the cardinal ligaments, posterior and anterior colporrhaphy, and perineal body fixation. The mesh was replaced inside the pelvis with the goal of reconstructing the tridimensional fascial disposition of the structures sustaining the correct axis of vagina. Follow-up was done at 12 months with POPIQ analysis. *Results*. One total mesh failure occurred early after surgery due to marked deficiency of anatomy. Two cystoceles were observed at 12 months in two patients treated for apical prolapse where anterior repair was not performed. Two other patients developed a de novo SUI at 12 months. No reported abnormalities of coital function or dyspareunia were ever found after surgery.* Conclusions*. It is possible that the utilization of a tension-free and tridimensional placement of Tutomesh might favor a more physiologic reconstruction of the vaginal axis as compared with traditional sacrospinous ligament suspension.

## 1. Introduction

Pelvic organ prolapse (POP) refers to loss of support of the anterior or posterior vaginal wall or the vaginal apex leading to protrusion into or out the vaginal canal of the bladder, rectum, small bowel, and uterus. In most cases POP is a collection of different support defects [1]. Among studies of ambulatory women the prevalence of POP is estimated to vary from 30% to 93% and the number of women seeking care for disorders of the pelvic floor is predicted to increase, looking at the population aging trends, by 45% in the near future [2]. The risk of POP increases with parity and advancing age but previous surgery to correct prolapse which almost always includes hysterectomy is the single greatest factor. In women whose initial hysterectomy was for genital prolapse the risk of repeating surgical correction has been estimated to recur in 20–43% of the cases [3]. Goals of surgery are many including relief of symptoms, correction of the anterior and posterior vaginal wall defects, prevention of new bladder or sexual problems, avoiding iatrogenic pelvic support defects, and achievement of long term success with no need for future pelvic surgery. Nevertheless because the success rate of initial surgery is depending on the capacity to restore normal pelvic floor anatomy, failure to address support defects at all levels is believed to predispose to recurrence of prolapse at the weakest point [1]. It is here briefly stated that the fibromuscular tissue of the vagina is enveloped by the endopelvic fascia which creates an anatomic and functional continuum with other ligaments including the cardinal/uterosacral ligaments (upper suspension level or level 1 DeLancey), lateral attachments to the arcus tendinous fascia and pubo-ileo-coccygeus fascia (middle suspension level or level 2 DeLancey), and at lower level the urogenital diaphragm anteriorly and perineal body posteriorly (lower suspension level or level 3 DeLancey) [4]. In addition to this the endopelvic fascia of the vagina also fuses anteriorly with the pubocervical fascia (Halban's fascia) and posteriorly with the rectovaginal fascia (Denonvilliers' fascia) [5, 6]. The pathophysiology of POP is multifactorial. It can result from genetic predisposition followed by defects of the connective tissue or muscular support or a combination. Tears in the endopelvic fascia permit the opposing soft tissues to bulge through the vaginal wall. Loss of muscular support places the endopelvic fascia under constant strain that results in damage to the connective tissue [1]. Tears in the endopelvic fascia can also cause stretch injury to the innervation of the muscular support. The insertion of the cardinal/uterosacral ligaments into the pericervical ring occurs at the level of the ischial spines and it is the detachment at this level that provides the anatomic rationale for development of posthysterectomy vaginal descent and enterocele (apical prolapse) [7, 8]. Surgery is limited to correction of connective tissue tears or breaks and overcorrection needs to be avoided because it can lead to new support problems. Although the uterus itself does not contribute to POP, most surgeons feel that removing the uterus maximizes the opportunity to correct apical support. There are many different surgical approaches to POP. They can be performed either abdominally or laparoscopically or vaginally [9–11]. Also they may imply the surgical employment of native tissue only or that of different sling or meshes grafts. Choice for best surgical correction of POP should be made on individual bases according to a number of different variables such as patient age, health conditions, and sexual activity as well as type of tissues defects [1]. For this reason different categories of operations exist best suited for various clinical indications having alternatively either obliterative or restorative or even compensatory finalities [1]. Here we describe a mixed restorative/compensatory surgical approach as a novel form of severe uterovaginal prolapse and vaginal vault descent treatment. The procedure involves the utilization of a new biological mesh of bovine pericardium (Tutomesh) for apical, lateral, and posterior fascial repair at the same time. For our purpose the xenograft mesh is precisely arranged and remodeled in our hands for multiple surgical landmarks sites affixation. These include deep and inward suspension to the right sacrospinous ligament, bilateral attachment to the cardinal ligaments remnants (paracervix and vaginal chorion), and forward anterior augmentation of native rectovaginal fascia (previously repaired) by means of final mesh attachment to the perineal body. By adopting such a technique an attempt is here made to ideally reproduce a tridimensional mesh replacement according to internal distribution of fascia and suspensory ligaments inside the pelvis (integral theory of Petros) [12]. Because of this tension-free mesh collocation, a lesser posterolateral deviation of the vaginal axis and also reduced apex narrowing are expected as compared to the traditional methods of sacrospinous ligament fixation. Although infections, when meshes are introduced vaginally, are always a possibility, the biological properties of such new mesh and the peculiar modality of its placement inside the pelvic floor may be of advantage to respect normal function of the surrounding tissues, reduce risk of tape erosion and dyspareunia, and at the same time maintain a lasting valid mechanical support of the pelvic organs.

## 2. Material and Methods

### 2.1. Patients

From October 2013 up to October 2014, thirty (*n* = 30) consecutive patients referred to our division for marked uterovaginal prolapse or posthysterectomy vaginal vault prolapse were treated. Informed consent was obtained from all patients scheduled for surgery for stage III or greater uterine prolapse (*n* = 20 patients) and stage II or greater vaginal cuff prolapse (*n* = 10 patients). Baseline assessments before surgery included a history and physical examination including POP-Q examinations on maximum Valsalva effort in the lithotomy position and urodynamic testing (Table 1). The POP-Q uses the hymen as a fixed point of reference and describes six specific topographic points on the vaginal wall (Aa, Ba, C, D, Bp, and Ap) and 3 distances (genital hiatus, perineal body, and total vaginal length). The prolapse of each segment is measured in centimeters during Valsalva relative to the hymenal ring with points inside the vagina reported as negative numbers and outside as positive [13]. The numeric values are then translated to a stage. Stage 3 refers to the lowest point > 1 cm below hymenal ring when the vagina is not completely prolapse. Also, condition-specific QoL and the effect of complications (i.e., protruding vaginal wall, dyspareunia, pain, voiding, and bowel dysfunction) were assessed using validated QoL questionnaires. Sexual life satisfaction and harmful effects of prolapse on QoL were determined by Prolapse Impact Sexual Questionnaire (PISQ-short form), the Urinary Impact Questionnaire (UIQ), the Colon Rectoanal Impact Questionnaire (CRAIQ), and the Pelvic Organs Prolapse Impact Questionnaire (POPIQ), respectively [14, 15]. The QoL instruments and the physical examination were repeated at 3 and 12 months after surgery. Standardized guidelines based on the literature and mesh manufacturer's recommendations for this specific surgical procedure were provided to all the surgical team participants. Only two surgeons have been involved in this study both of them with experience in pelvic surgery.

### 2.2. Mesh (Tutomesh): “Butterfly 3D” (Kit Patented)

**Figure 1:**
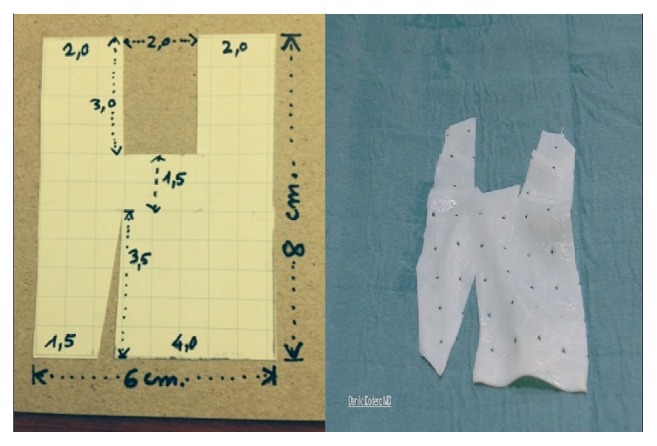


**Figure 2:**
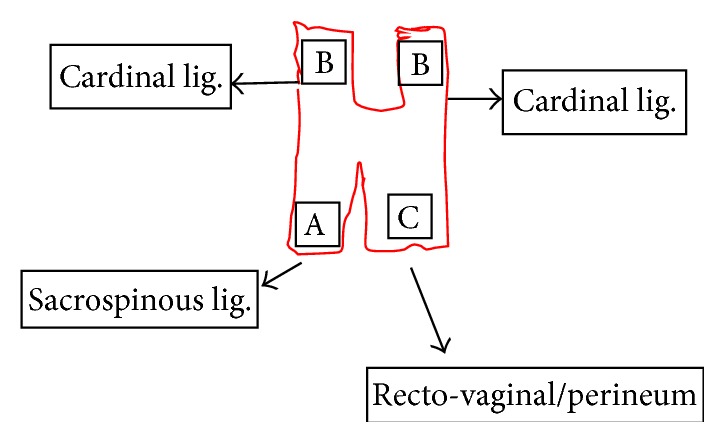


**Figure 3:**
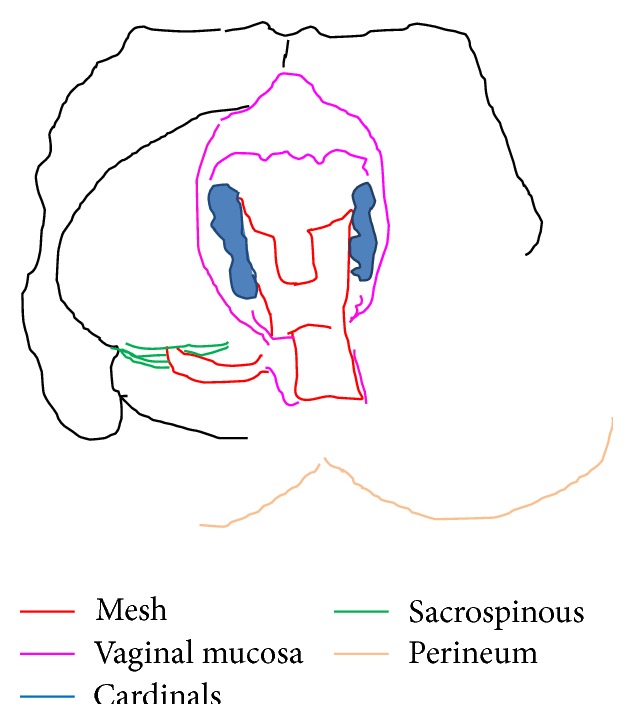


Tutomesh^©^ is an avital, acellular, xenogeneic collagen membrane made from bovine pericardium meeting the standards of safety and quality, for instance, according to the German Medical Device Act (MPG) or according to the European Directives (93/42/EEC and 2003/32/EC). The raw material exclusively originates from BSE-free countries and is subjected to the proven Tutoplast process. Tutomesh consists of 92% native collagen type I, which is maintained in its three-dimensional structure and for its biomechanical properties. This renders the transplant extremely resistant to tensile forces without impeding the remodeling process after implantation. Tutomesh is a natural collagen membrane without further treatment for cross-linking. If handled and placed appropriately the mesh acts as a scaffold which allows the in-growth of vessels and fibroblasts with their deposition of site-specific collagen. In this way Tutomesh is gradually replaced by the patient's own tissue which will transform into the site-specific tissue by time. For the fixation of Tutomesh monofilament and not resorbable suturing material is recommended. In order to prevent the suture from cutting the mesh, the edge of the Tutomesh should be folded in for approximately 1 cm [16, 17]. Clinical practice suggests that the mesh is perfectly positioned by fixing it at multiple points with interrupted sutures. For our purposes Tutomesh is retailed in H shaped form in order to be attached to multiple surgical landmarks, avoiding direct tension and deviation of the vaginal axis at the same time: A, sacrospinous ligament; B, cardinal ligaments; C, perineal body (Figures 1, 2, and 3).

### 2.3. Surgical Procedure

At our division vaginal hysterectomy is performed from a long time based on classic principles of vaginal surgery with recent modifications due to the employment of modern surgical instruments and precious teachings personally received at the Emory University Hospital (USA) by Professor Kovac [18]. A number of almost 200 vaginal hysterectomies are performed annually most for grade I or II uterine-vaginal prolapse frequently coupled to anterior and/or posterior colporrhaphy. In general when vaginal hysterectomy is performed for mild uterovaginal prolapse, our routine practice is to add a prophylactic modified McCall culdoplasty at the end of hysterectomy [19–21]. To this end the vaginal vault is closed by executing a ligamentous form of colpopexy as follows: one end of a suture previously inserted in the anterior flap of peritoneum is used for taking successive bites in the peritoneum on the right side until the suture reaches the right broad ligament where it is passed through the tuboovarian stump. The suture picks up the peritoneum and the broad ligament in successive bites and includes the stumps of the uterine vessels and uterosacral ligament. Care is taken to insert the needle distal to the ligatures. The suture is then passed through the right edge of the posterior peritoneal flap, through the posterior vaginal wall and out into the right side of the posterior vaginal fornix. A similar suture begun on the anterior portion of the peritoneum is placed on the left side to close the left half of the open vaginal vault. These sutures are then separately tied. These sutures anchor the broad and uterosacral ligaments to the vaginal vault, take care of peritonization, and close the vaginal vault. The anterior and posterior vaginal walls are approximated with interrupted or continuous sutures (Vicryl 0, Ethicon, Johnson & Johnson, USA). A cystocele or rectocele is repaired at this time.

In cases of worse uterovaginal prolapse we have always traditionally recurred to restorative (sacrospinous ligament suspension) or even compensatory (total vaginal wall mesh repair) forms of surgical repair according to Amreich-Richter or Prolift (Gynecare Prolift, Ethicon, Johnson & Johnson, USA) recommendations, respectively. Sometimes the two approaches have been also variably combined. Despite the overall good results achieved in most cases with both these approaches, our anecdotal experience of some complications and recurrences (in the percentage commonly reported for these techniques) prompted us toward the research for a possibly better way of repair as here described in detail.

*(A) Vaginal Hysterectomy for Grade III Uterine-Vaginal Prolapse.* First an infiltration of the anterior, lateral, and posterior vaginal fornix is made by using a modified physiologic solution (100 cc of saline solution + 30 IU of Oxytocin + 2 ampules of Naropin 2 mg/mL). With a scalpel a transverse incision is made through the anterior vaginal mucosa below the attachment of the bladder and prosecuted by less deep lateral incision on each side of the cervix toward the posterior vaginal fornix which is incised at a considerable distance from the external os. The anterior wall of the vagina with the attached bladder is then separated from the uterus with bipolar scissors. Afterwards the incised posterior mucosa is pushed down and back to expose the peritoneum. The posterior cul-de-sac peritoneum is opened with scissors and a narrow retractor inserted into the peritoneal cavity. The description of the vaginal hysterectomy is familiar to every surgeon and it is not further described here because it is outside of the study scope. Anterior colporrhaphy has been always executed in all cases (*n* = 20). A vertical incision of the vagina is made in the midline. The vaginal muscularis is plicated using 2-0 absorbable suture in an interrupted fashion (Vicryl 2-0, Ethicon, Johnson & Johnson, USA). Care is taken to ensure that the vaginal muscularis is not removed from the underlying detrusor muscle. If there is loss of the urethrovesical angle a plicating suture at the urethrovesical junction is placed to restore anatomy (the Kelly plication). Sometimes one bridge suture passing through the pubourethral ligaments on each side (Nichols suspension) or a transobturator mesh urethroplasty (TOT) is alternatively performed to correct urethral mobility and prevent de novo IUS occurrence [16]. The redundant vaginal wall is then resected and the vaginal edges are reopposed using 2-0 absorbable suture in a continuous crossed fashion while being careful to pick up underlying vaginal muscularis to close the dead space (Vicryl 2-0, Ethicon, Johnson & Johnson, USA).

*(B) Vaginal Vault Prolapse*. Two Allis clamps are placed at the recognizable angles of the vaginal scar. The saline solution is similarly injected under the vaginal mucosa followed by a vertical or transverse incision of the mucosa. Using scissors and toothed pickups the vaginal epithelium including the muscularis is dissected with caution from the underlying peritoneum (enterocele) and bladder or rectum mucosa. Care is taken to respect integrity of the fragile peritoneal folds below the dissection. Anterior colporrhaphy to reduce the protrusion of the bladder and vagina in order to prevent cystocele recurrence is generally performed. However, in the series here studied this was done only in 8 out 10 patients. 

**Figure 4:**
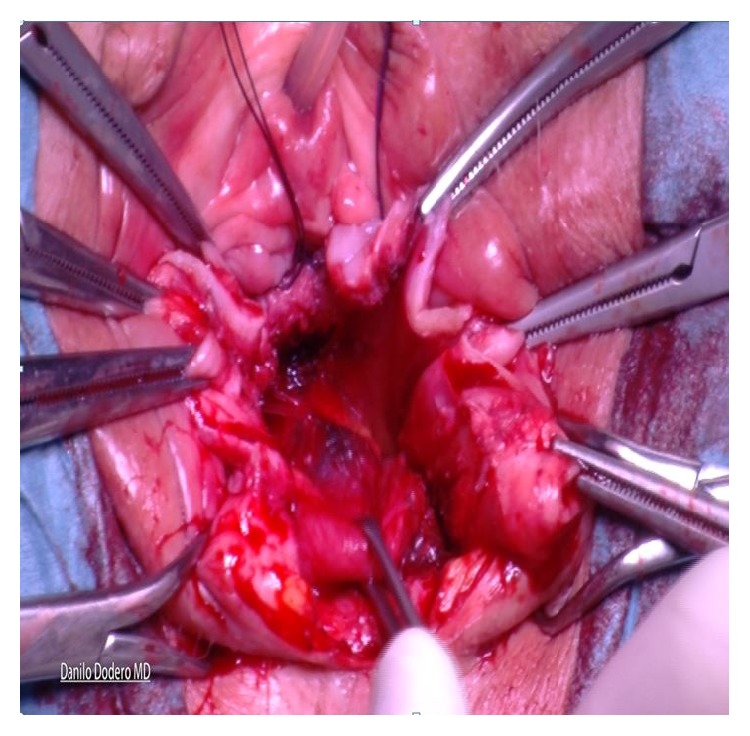


*(C) Posterior Compartment Repair (Same for A and B)*. Posterior colporrhaphy includes the plication of the pararectal and rectovaginal fascia over the rectal wall. In the past, levator ani plications used to be more frequently done for the treatment of rectocele but because of dyspareunia we moved toward a fascial plication only according to Kovac [18]. Posterior compartment repair is similar, done either after vaginal hysterectomy or in presence of apical vault prolapse: after injecting the saline solution under the vaginal mucosa, a longitudinal posterior colpotomy is performed in the midline after a diamond-shaped incision of the perineum. Once again the vaginal epithelium is carefully dissected from the muscularis (rectovaginal fascia) and left apart along with the rectum wall. The pararectal space is bilaterally prepared with mobilization of the rectum from its lateral connections by blunt finger dissection. After introducing one finger into the rectum it is possible to better evaluate size and level of fascial defect and provide best fascial repair. After clamping with Allis the rectovaginal fascia (Figure 4), interrupted sutures to close the defect and anchor the rectovaginal fascia to the perineal body are given. During this suture a finger inside the rectum allows checking for eventual mistaken bites of the rectal mucosa into the suture.

**Figure 5:**
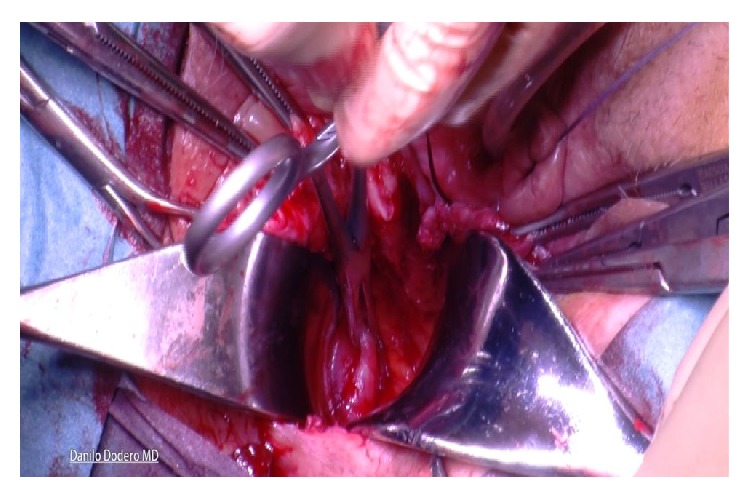


The procedure used for sacrospinous fixation is basically done according to the technique originally described by Amreich-Richter [22, 23] although having here a different rationale. The technique comprises dissection into the right paravaginal space, identification of the right ischial spine, and clear visualization of the right sacrospinous ligament (Figure 5).

**Figure 6:**
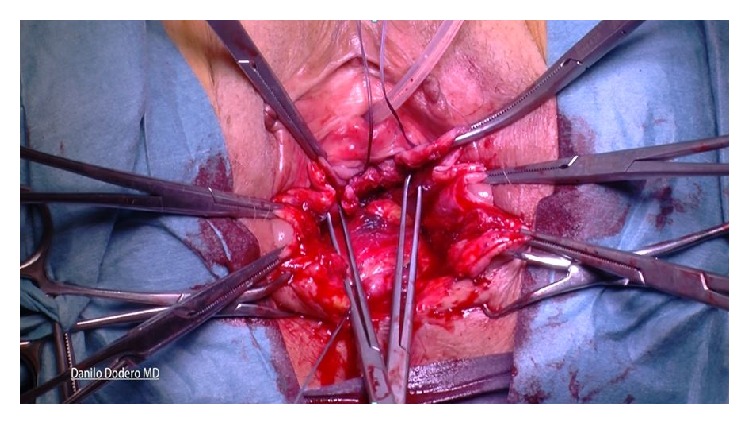


**Figure 7:**
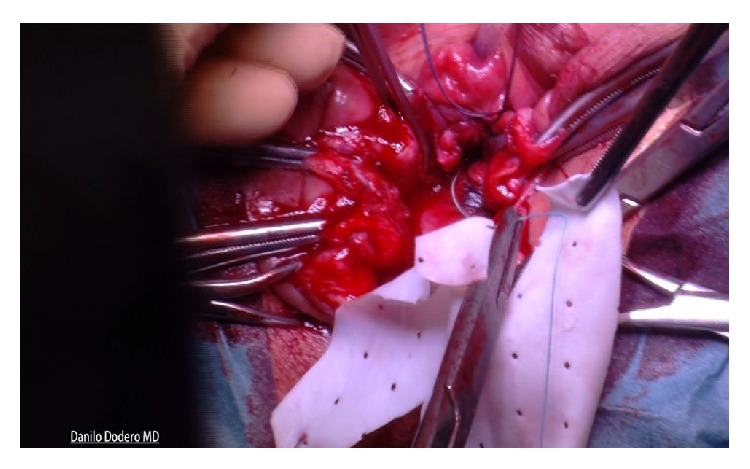


Using one single no absorbable 2-0 thread a double stitched suture (Ethibond Excel 2-0, Ethicon, Johnson & Johnson, USA) is placed through the ligament one and half fingerbreadths from the tip of the ischial spine. Both ends of the suture are left untied and kept for later transfixion of the inferior right arm of the H shaped Tutomesh (“Butterfly 3D” kit patented). In our technique whatsoever direct attachment of the vaginal vault to the ligament is intentionally avoided. Next step implies the recognition of the cardinal ligaments which are generally found as thick paracervical remnants or parts of the vaginal chorion on each side. Cardinal ligaments are then held by Allis clamps in view of delayed transfixion with no adsorbable 2-0 sutures to the upper arms of the H shaped Tutomesh (Figures 6 and 7).

**Figure 8:**
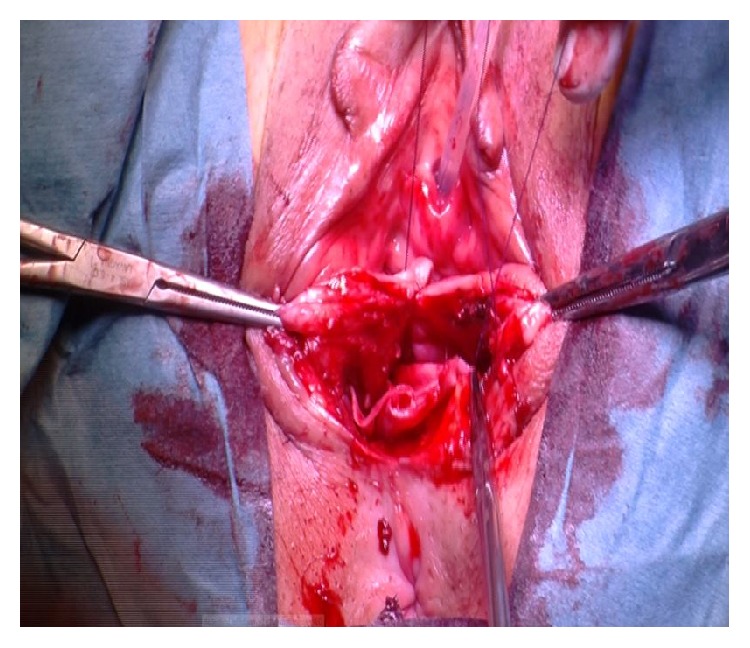


**Figure 9:**
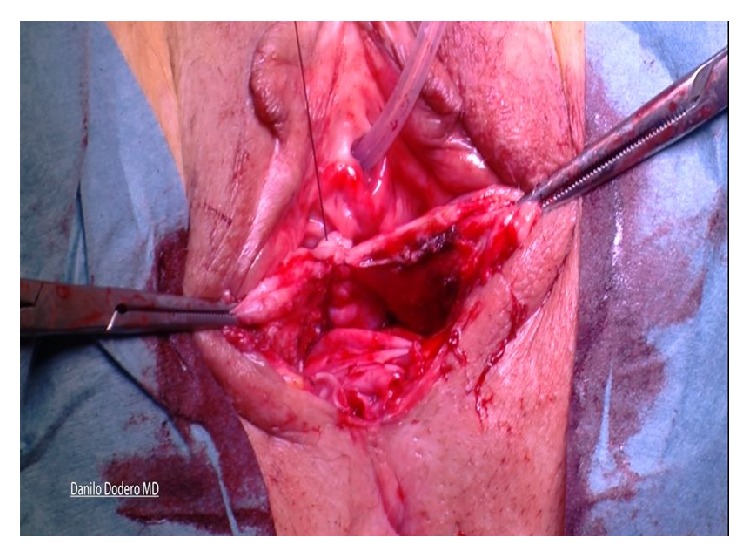


As soon as all the surgical landmarks are localized and exposed, the Tutomesh graft, previously adequately molded and cut, is then placed in situ. After suturing the vaginal apex with interrupted stiches of Vicryl 0, the posterior colporrhaphy is started using Vicryl 2-0 in a continuous not crossed fashion. When the colporrhaphy reaches the mid portion of the vagina it is halted. The four Tutomesh arms can be now tied as follows: the inferior right arm is sutured to the sacrospinous ligament, the two upper lateral arms are sutured to the cardinal ligaments, and the inferior left arm is sutured to the perineal body (Figures 8 and 9).

**Figure 10:**
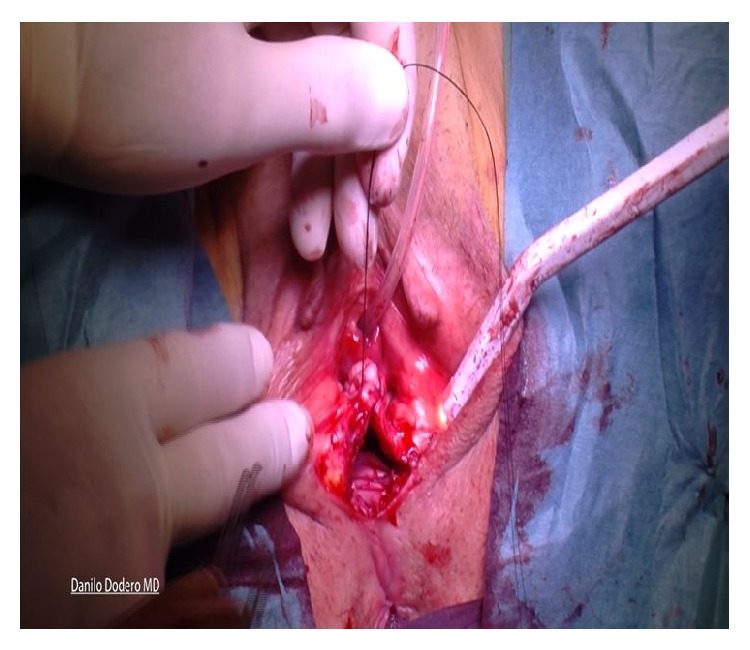


In this last case a barbed suture (Quill 2-0, Surgical Specialties Corporation, USA) in a continuous fashion is generally done. Thereafter the colporrhaphy is completed ending with final cosmetic repair of the perineum (Figure 10). Iodine gauze is left in vagina for 24 hours and urinary catheter kept in place for 72 hours. On the day of surgery, cefazolin 2 g was administered. Prophylactic anticoagulant therapy was given for up to 28 days. All patients are advised to start and prosecute for at least 1 month a local estrogen therapy by vaginal route.

## 3. Results

Operative parameters and short term complications are reported in Table 2. The median operation time was 90 minutes (range 55–166 minutes) and median blood loss was 120 mL (20–790 mL). No patients required a blood transfusion. Importantly, one case of early total surgical failure due to suboptimal suspension to the sacrospinous ligament in a 60-year-old patient with apical prolapse was observed. In this case the visualization and transfixion of the ligament during the intervention resulting were particularly difficult because of paucity and laxity of ischiorectal tissues. This patient was readmitted later soon and reoperated on by means of abdominal sacrocolpopexy. Additional prolapse recurrence occurred in two cases (cystocele) out of 30 and specifically involved only those 2 cases of apical prolapse where a prophylactic repair of the anterior compartment was inadvertently not done. This was observed at 12-month interval. However, only one patient in this subgroup was symptomatic and required reoperation by anterior colporrhaphy and transobturator mesh urethroplasty (TOT). Finally, in other two patients a de novo urinary stress incontinence at 3 months after hysterectomy was present requiring, again, a TOT operation. Overall in 4 out of 30 cases (13.3%) it was necessary to repeat surgery.

**Figure 11:**
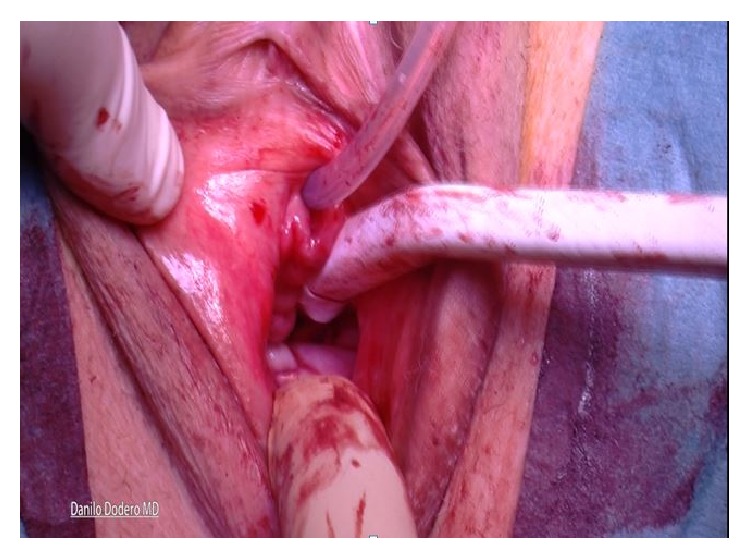


A normalization of the vaginal axis with satisfactory length and width was achieved in all cases with importantly no report of dyspareunia or hyspareunia (male partner complaints due to vaginal axis narrowing as it occurs when one vaginal cuff corner is fixed tightly to the sacrospinous ligament) (Figure 11). Mean total vaginal length was 8.2 cm and the mean point C position as a measure of vaginal apex fixation above the hymen, one year after surgery, was −4.99 (Table 3). A significant improvement in the quality-of-life questionnaires after surgery was found in most cases (Table 4).

## 4. Discussion

Treatment and prevention of vaginal vault prolapse are challenging as they are shown by the existence of more than 40 different techniques to treat this pathology. Moreover controversy also exists over the choice of vaginal procedure as well as the relative merits of vaginal versus abdominal suspension procedures. In general, for elderly patients whose health status precludes prolonged surgery, an obliterative repair closing vagina and affording symptom relief with minimal morbidity is preferred (colpocleisis) while, for those patients with discrete defects in the endopelvic fascia without ongoing risk factors for recurrence, a restorative procedure accomplished by a vaginal approach is rather favored (sacrospinous ligament suspension or iliococcygeus fascial suspension or uterosacral ligament suspension in variable combination with anterior colporrhaphy or paravaginal repair and posterior colporrhaphy). In other circumstances the native tissue repair however is insufficient and a compensatory operation using grafts materials (meshes) became a more reasonable option (abdominal or laparoscopic sacral colpopexy or anterior and posterior total vaginal wall mesh replacement or infracoccygeal IVS sling colpopexy) [1–3, 5–7]. Nonetheless, graft materials, particularly when used by a vaginal approach, may shrink after placement (mesh erosion) or lead to loss of pelvic floor flexibility (dyspareunia) or be site of late infections [24, 25]. For this reason a novel laparoscopic technique employing only native tissues such as the obliterated umbilical arteries anchored to the vaginal cuff (laparoscopic chordofixation) has more recently been proposed as a safer and faster option [26]. Notwithstanding the above, no technique is as yet fully satisfactory reflecting how poorly the pathophysiology is understood and so its correction [27]. In addition, the choice of procedure is often dependent on the individual surgeon's choice and experience.

Our long time experience of vaginal surgery has allowed addressing an original strategy for the correction of advanced uterine-vaginal prolapse in order to prevent it (at the time of vaginal hysterectomy) and directly cure it as well. We hypothesized that the use of a new biological mesh for a compensatory repair in combination with a restorative one (sacrospinous ligament-mesh fixation) could possibly be of advantage if compared with total wall synthetic mesh repair or sacrospinous ligament suspension as separately considered. Our procedure allows replacing fascial defects with a biological mesh of bovine pericardium properly arranged for being anchored to the sustaining connective structures and ligaments which naturally follow a tridimensional way of disposition and support inside the pelvic floor. Importantly, level I DeLancey apex suspension is obtained indirectly by fixing the mesh to the vaginal chorion and paracervical tissue containing the cardinals and only secondly by suspending the mesh to the right sacrospinous ligament. We believe this point to be a striking feature of our procedure since it reduces a lot the vaginal tension and posterolateral axis deviation as usually reported in case of traditional procedure for sacrospinous ligament fixation. By suturing the mesh also to the perineal body (just over the previously repaired rectovaginal fascia) and completing the operation with an anterior colporrhaphy it is possible at the end to obtain a full restitutio ad integrum of the vaginal support with good urinary continence, vaginal capacity, and coital function. The level I vaginal suspension so executed along with the anterior and posterior fascial and ligamentous repair also would reinforce the pelvic floor thus making unlikely the formation of tears or breaks of the pelvic floor conducing to recurrence. Our results show this to be the case. In fact the only three cases with unsuccessful outcome should be ascribed in one case to an unpredictable severe macroscopic (and probably microscopic) deficiency of the connective tissue of the ischiorectal space (one total failure) and in the other two cases to the missed anterior compartment repair at the time of apical vault surgery (two cystoceles). The cystocele recurrence risk deserves particular and separate consideration whenever a solid apex fixation and efficient posterior reinforcement, such as here done, are provided.

To our knowledge this is the first time that a biological mesh of bovine pericardium (Tutomesh) is used in gynecological surgery even though we took into account the same surgical principles already reported using Tutomesh in case of abdominal wall hernias repair [17]. Tutomesh appears to greatly satisfy many of the requests for a prosthetic graft to be ideal including biocompatibility, inert activity, no allergic or inflammatory reaction, and sterility, no carcinogen, and also handling feasibility. However, despite the significant increase in the use of biological products to overcome the problems of prosthesis erosion and extrusion it is generally still an open question of how long any biological absorbable material has to remain as a scaffold (and not only as bridge) before adequate in-growth of host tissues has occurred to maintain long term support [28]. Up to date whether, among a number of new absorbable and biological meshes proposed, Tutomesh might be more resistant to infections or be particularly suited for avoiding shrinkage and mechanical stress while preserving its intrinsic strength has still to be determined.

In any case, we believe that our procedure is original since we have not been able to find anything similar in the literature.

We refer to the review article of Birch on the use of prosthetics in pelvic reconstructive surgery [28]. In this review two studies using a similar combination of restorative/compensatory surgery (for the cure of separate prolapse compartments) have been reported. Salomon et al. [29] described the use of porcine skin collagen (Pelvicol) introduced through a transobturator approach in combination with a sacrospinous fixation for the anterior compartment repair. Their short term results were encouraging. Dwyer and O'Reilly described the fixation of a Y-shaped polypropylene prosthesis to the sacrospinous ligament bilaterally and an inferior attachment to the perineal body for the posterior compartment repair. In their study of 33 patients, erosions were reported in 4% with no reports of postoperative sexual dysfunction [30].

In conclusion, we have here described a new approach aimed at restoring, in case of severe POP, the first, the third, and only partially the second (posterior half) of DeLancey levels. Despite our encouraging preliminary results here reported using the “Butterfly 3D” Tutomesh (low POP recurrence, no mesh erosions, and no dyspareunia), due to the limited number of cases studied and short follow-up period we cannot, to date, draw firm long term conclusions. At the moment, we are now in the process of extending our studies in a multicenter randomized trial (protocol BioPOP approved by Regional Ethic Committee) involving 15 centers in Italy to enroll at least 300 patients as for adequate statistical power analyses request. At the same time we have initiated a study on fresh cadavers to evaluate the possibility to correct completely also the second DeLancey level by means of a new biological mesh of different size (ASSUT Spa).

## Figures and Tables

**Table 1:** Patients characteristics: sample means and SDs.

Number of patients	30
Number of cases with uterine prolapse stage III or IV	20
Number of cases with apical prolapse	10
After vaginal hysterectomy for prolapse	7
After vaginal hysterectomy	2
After abdominal hysterectomy	1
Age (SD)	68.41 (9.22)
Body mass index (SD)	28.11 (3.9)
Parity (SD)	2.5 (0.50)

**Table 2:** List of operative parameters and postoperative complications at 1 month.

Variable	With hysterectomy	Without hysterectomy
Number of patients	20	10
Hemoglobin drop	2.0 ± 1 g%	1.5 ± 1 g%
Bladder lesions	0	0
Hematoma	0	1 (drained)
Abscess	2 (spontaneous resolution)	0
Bowel lesions	0	0
Concomitant pelvic surgery	Bilateral oophorectomy in 12 cases	0

**Table 3:** POP-Q findings.

	Before surgery	3-month follow-up	12-month follow-up
Aa	1.5	−2	−2.31
Ba	2	−1.9	−2.13
C	1.5	−5.0	−4.99
Gh	5.2	4.2	3
Pb	4.1	4.5	3.44
Ap	1.1	−2.13	−2.37
Bp	1.2	−2.1	−2.22

**Table 4:** Quality-of-life questionnaires.

Months after surgery	PISQ-12	UIQ	CRAIQ	POPIQ
0	28.9	100.22	36.86	76.70
3	33.2	59.11	15.88	16.15
12	36.7	31.7^*∗*^	4.61^*∗*^	10.2^*∗*^

^*∗*^ *p* value <0.001 in the score between months 12 and 0. PISQ-12: pelvic organ prolapse urinary incontinence sexual function questionnaire-12, UIQ: Urinary Impact Questionnaire, CRAIQ: Colon Rectoanal Impact Questionnaire, and POPIQ: Pelvic Organs Prolapse Impact Questionnaire.

## References

[1] Rojas F., Cundiff G. W. (2007). Urogynecology and reconstructive pelvic surgery. *The John Hopkins Manual of Gynecology and Obstetrics*.

[2] Alperin M., Weinstein M., Kivnick S., Duong T. H., Menefee S. (2013). A randomized trial of prophylactic uterosacral ligament suspension at the time of hysterectomy for Prevention of Vaginal Vault Prolapse (PULS): design and methods. *Contemporary Clinical Trials*.

[3] Blandon R. E., Bharucha A. E., Melton L. J. (2007). Incidence of pelvic floor repair after hysterectomy: a population-based cohort study. *The American Journal of Obstetrics and Gynecology*.

[4] DeLancey J. O. L. (1992). Anatomic aspects of vaginal eversion after hysterectomy. *American Journal of Obstetrics and Gynecology*.

[5] Chaliha C., Khullar V. (2005). Management of vault prolapse. *Reviews in Gynaecological Practice*.

[6] Afifi R., Sayed A. T. (2005). Post-hysterectomy vaginal vault prolapse. *The Obstetrician & Gynaecologist*.

[7] McCracken G. R., Lefebvre G. (2007). Mesh-free anterior vaginal wall repair: history or best practice?. *The Obstetrician & Gynaecologist*.

[8] Uzoma A., Farag K. A. (2009). Vaginal vault prolapse. *Obstetrics and Gynecology International*.

[9] Arbel R., Lavy Y. (2005). Vaginal vault prolapse: choice of operation. *Best Practice and Research: Clinical Obstetrics and Gynaecology*.

[10] RCOG (2007). The management of post hysterectomy vaginal vault prolapse. *Green-Top Guideline*.

[11] Khunda A., Vashisht A., Cutner A. (2013). New procedures for uterine prolapse. *Best Practice & Research: Clinical Obstetrics & Gynaecology*.

[12] Petros P. (2010). *The Female Pelvic Floor: Function, Dysfunction and Management According to the Integral Theory*.

[13] Bent A. E., Ostegard D. R., Cundiff G. W., Swift S. (2003). *Ostergard's Urogynecology and Pelvic Floor Dysfunction*.

[14] Halaska M., Maxova K., Sottner O. (2012). A multicenter, randomized, prospective, controlled study comparing sacrospinous fixation and transvaginal mesh in the treatment of posthysterectomy vaginal vault prolapse. *The American Journal of Obstetrics and Gynecology*.

[15] Shull B., Karram M. M. (2005). Concerns regarding pelvic reconstructive surgery. *International Urogynecology Journal and Pelvic Floor Dysfunction*.

[16] Prodigalidad L. T., Peled Y., Stanton S. L., Krissi H. (2013). Long-term results of prolapse recurrence and functional outcome after vaginal hysterectomy. *International Journal of Gynecology and Obstetrics*.

[17] D'Ambra L., Berti S., Feleppa C., Magistrelli P., Bonfante P., Falco E. (2012). Use of bovine pericardium graft for abdominal wall reconstruction in contaminated fields. *World Journal of Gastrointestinal Surgery*.

[18] Kovac S. R. (2014). Route of hysterectomy: an evidence-based approach. *Clinical Obstetrics and Gynecology*.

[19] Cruikshank S. H., Kovac S. R. (1999). Randomized comparison of three surgical methods used at the time of vaginal hysterectomy to prevent posterior enterocele. *American Journal of Obstetrics and Gynecology*.

[20] Cruikshank S. H. (2009). Operations for support of the vaginal vault. *The Global Library of Women's Medicine*.

[21] Nichols D. H., Randall C. L. (1989). *Vaginal Surgery*.

[22] Randall C. L., Nichols D. H. (1971). Surgical treatment of vaginal inversion. *Obstetrics and Gynecology*.

[23] Cruikshank S. H. (1991). Sacrospinous fixation—should this be performed at the time of vaginal hysterectomy?. *American Journal of Obstetrics and Gynecology*.

[24] Biller D., Davila W. G. (2004). Choosing the best technique for vaginal vault prolapse. *OBG Management Journal*.

[25] Iglesia C. B. (2013). STOP using synthetic mesh for routine repair of pelvic organ prolapse. *OBG Management Journal*.

[26] Koppan M., Hackethal A., Muller-Funogea I. A. (2012). Laparoscopic chordofixation: a new technique for vaginal vault suspension. *Pelviperineology*.

[27] Lukanovič A., Dražič K. (2010). Risk factors for vaginal prolapse after hysterectomy. *International Journal of Gynecology and Obstetrics*.

[28] Birch C. (2005). The use of prosthetics in pelvic reconstructive surgery. *Best Practice & Research: Clinical Obstetrics and Gynaecology*.

[29] Salomon L. J., Detchev R., Barranger E., Cortez A., Callard P., Darai E. (2004). Treatment of anterior vaginal wall prolapse with porcine skin collagen implant by the transobturator route: preliminary results. *European Urology*.

[30] Dwyer P. L., O'Reilly B. A. (2004). Transvaginal repair of anterior and posterior compartment prolapse with Atrium polypropylene mesh. *BJOG: An International Journal of Obstetrics & Gynaecology*.

